# Expression of ezrin is associated with invasion and dedifferentiation of hepatitis B related hepatocellular carcinoma

**DOI:** 10.1186/1471-2407-9-233

**Published:** 2009-07-15

**Authors:** Chun-Nan Yeh, See-Tong Pang, Tsung-Wen Chen, Ren-Ching Wu, Wen-Hui Weng, Miin-Fu Chen

**Affiliations:** 1 Department of Surgery, Chang Gung Memorial Hospital; Chang Gung University, Taoyuan, Taiwan, Republic of China; 2 Department of Urology, Chang Gung Memorial Hospital; Chang Gung University, Taoyuan, Taiwan, Republic of China; 3 Department of Pathology, Chang Gung Memorial Hospital; Chang Gung University, Taoyuan, Taiwan, Republic of China; 4 Department of Chemical Engineering and Biotechnology, National Taipei University of Technology, Taipei City, Taiwan, Republic of China

## Abstract

**Background:**

Hepatocellular carcinoma (HCC) is the fifth most common malignancy in the world and constitutes the leading cause of cancer-related death among men, and second among women in Taiwan. Liver cirrhosis and HCC are relatively prevalent, and 80% to 85% of the patients with these conditions have positive results for hepatitis B surface antigen in Taiwan. Only 5% of the general population is seronegative for all hepatititis B virus (HBV) markers. This is the first study to determine the role of ezrin upon HBV HCC cell and patients with HBV HCC undergoing hepatectomy

**Methods:**

Immunohistochemical study with ezrin in 104 human HBV-HCC cases were carried out to investigate its association with the clinicopathological features and the outcomes of 104 HBV-HCC patients undergoing hepatetomy. In addition, DNA constructs including the wild type ezrin (wt-ezrin) and mutant ezrin Tyr353 (Y353) were transfected into Hep3B cell to study its role in tumor invasion and differentiation.

**Results:**

HBV HCC patients with ezrin over-expression independently have smaller tumor size, cirrhotic liver background, poor tumor differentiation, and more vascular invasion. Ezrin expression status has no impact on survival for HBV-HCC patients undergoing hepatectomy. The in vitro assay showed that wt-ezrin Hep3B cells have a significant higher level of AFP secretion and higher invasion ability as compared with the control and Y353- ezrin Hep3B cells.

**Conclusion:**

Ezrin over-expression contributed to de-differentiation and invasion of HBV-HCC cell. HBV-HCC patients with ezrin over-expression were independently associated with tumor with smaller size, cirrhotic liver background, poor differentiation, and vascular invasion.

## Background

Hepatocellular carcinoma (HCC) is a common disease in Taiwan, with an age-adjusted incidence of 28.7 per 100000 of the population per annum. It continues to be the leading cause of cancer-related death among men and the second among women [1]. In Taiwan, liver cirrhosis and HCC are relatively prevalent, and 80% to 85% of the patients with these conditions have positive results for hepatitis B surface antigen (HBsAg) [2-4]. Only 5% to 6% of the general population is seronegative for all HBV markers [2-4]. Our previous study revealed the positive rates of HBsAg and hepatitis C virus antibody were 61.9% and 35.3%, respectively, in patients with HCC undergoing hepatic resection [5].

The recent identification of ezrin, the product of the Villin 2 gene, as a crucial molecule in the dissemination of two pediatric tumors adds new protein to the list of metastasis-associated molecules [6,7]. Ezrin is a member of the ERM (ezrin-radixin-moesin) cytoskeleton-associated protein family [8], sharing a homology with the amino-terminal membrane-binding domain of erythrocyte band 4.1 and possessing membrane-cytoskeleton linking functions [9]. Ezrin plays a positive role in maintaining cell shape and polarity and participates in cell migration, signaling, growth regulation, and differentiation [10]. Ezrin can interact with several membrane proteins, including CD44 [11], CD43 [11], intercellular adhesion molecule-1 and intercellular adhesion molecule-2, and phosphatidylinositol [4,5]-bisphosphate [12]. In addition, ezrin can signal cell survival through the phosphatidylinositol 3-kinase/Akt pathway [13]. Ezrin is also actively involved in regulating the growth and metastatic capacity of cancer. Over-expression of ezrin has been detected in several human epithelial tumors, including prostate cancer [14,15], brain hemangioblastoma [16], uterine endometrioid adenocarcinoma [17], osteosarcoma [18], and uveal malignant melanoma [19].

However, the role of ezrin on HBV-HCC is unknown. The aim of this study was to help to determine the role of ezrin upon patients with HBV HCC undergoing hepatectomy in terms of demographics, laboratory data, operative findings, pathological features, and survival. In addition, in vitro study using DNA constructs including the wild type ezrin (wt-ezrin) and Tyr353 mutant ezrin (Y353-ezrin) were transfected into Hep3B cell to study its role in tumor invasion and differentiation.

## Methods

### Clinicopathological features of HBV HCC patients

From 1985 to 2001, 104 HBV HCC patients undergoing hepatectomy at department of surgery, Chang Gung Memorial Hospital, Taipei, Taiwan were investigated. Laboratory tests were performed on the day before surgery. Child grading was used to classify the severity of cirrhosis of HBV HCC patients. *The study was approved by the local institutional review board (IRB) of Chang Gung Memorial Hospital in the genetic transfection and clinical study and all patients gave informed consent before taking part in the study for immunostaining (No: 94-955B)*. Histopathological findings of HCC were divided into four grades according to Edmondson and Stainer's system [20]. Grades I and II were conditioned as low-grade HCC, and grades III and IV as high-grade.

### Follow-up study

Follow-up evaluation involved clinical physical examinations and blood chemistry tests at each visit. The remnant liver was examined using abdominal ultrasonography (US) every three months, and the serum α-fetoprotein (AFP) was measured by radioimmunoassay at least every 3 months. When US detected a new lesion, or elevated AFP was noted, an abdominal CT, MRI, or liver scan was performed for confirmation. Chest x-ray examination was performed every 6 months for pulmonary metastasis evaluation. If a patient complained of bone pain, a bone scan was performed for the detection of metastasis. If any of the above procedures indicated recurrence, the patient was readmitted for further investigation, including an angiographic evaluation or MRI.

### Cell culture

Hep 3B, a stable hepatitis-B related human HCC cell line containing an integrated hepatitis B virus genome, was routinely maintained in DMEM medium. This medium contains 5 mg/mL phenol red and supplemented with 10% fetal bovine serum (FBS), 2 mM L-glutamine and 50 mg/mL each of penicillin and streptomycin (all purchased from Life Technologies Ltd, Paisley, UK).

### Mutagenesis and cell transfection

An expression construct with VSV-G-tagged human wild type ezrin (wt-ezrin) and Y353 mutant ezrin were a kindly provided by Professor Monique Arpin [13]. Hep 3B cell was seeded at a density of 1.5 × 10^6^/60 mm dish in 2 ml of DMEM medium one day before transfection. Transfection of ezrin expression constructs was performed using Lipofectamine 2000 reagent (Invitrogen, Carlsbad, CA) according to the manufacturer instructions. Neomycin was added into the culture medium for selecting positively transfected clones.

### Western Blotting

Hep 3B cell was cultivated and treated as described above. The cells were harvested using RIPA buffer with protease inhibitor. Protein concentration was determined with BCA Protein Assay Reagent (Pierce, USA). Equal amounts of protein were denatured with loading buffer, separated on a 12% SDS-PAGE gel (Invitrogen Life Technologies, UK) and blotted onto a PVDF membrane (Amersham, Life Science, UK). Membranes were then blocked in a Tris-buffered saline solution with 5% BSA or nonfat dry milk and probed with primary antibody. The membranes were further probed with HRP-conjugated goat anti-mouse/anti-rabbit to visualize band. Mouse monoclonal anti-human ezrin antibody was obtained from company (Ab-1; Neomarker, Lab Vision, CA, USA) and mouse anti-VSV-G antibody was obtained from Roche.

### Matrigel invasive assay

Matrigel was purchased from Becton Dickinson (San Jose, CA, USA) and stored at -20°C. After thawing at 4°C overnight, matrigel was diluted in serum free-cold DMEM medium and 50 ul were evenly inoculated onto upper chamber of 6.5 mm transwell membrane and allowed to form a gel at 37°C incubator. The Matrigel invasive assay was carried out according to the manufacturer's instructions. A 0.2 mL of aliquot of cell suspension containing 3.3 × 10^5 ^cells was seeded on the upper chamber of Matrigel coated transwell filter (8 um pore). The serum-containing medium was added to the lower chamber and incubated for 48 hrs at 37°C in a humidified atmosphere of 5% CO_2_. Non-invading cells that remained on the upper surface of the filter were removed with cotton wool; the cells that appeared on the lower surface of the filter were fixed in 4% paraformaldehyde, stained with hematoxylin and counted under a microscope. Each assay was carried out in triplicate in three different occasions.

### Differentiation assay

Detection of alpha-fetoprotein by radioimmunoassay and albumin by biochemical test: 5 × 10^6 ^cells in 10 ml medium were plated in 100 mm culture plate. Incubate cells at 37°C in a CO_2 _incubator for 40 hours. The supernatant was harvested for test and cell number was counted for adjustment.

### Immunohistochemistry

From the archives of Chang Gung Memorial Hospital (from 1985 to 2001), each case of HCC was selected based on the availability of sufficient quantities of tumor cells. H&E-stained slides from each case were reviewed. 4 μm section of 104 patients with HBV related HCCs from formalin-fixed, paraffin-embedded tissue were stained for ezrin. The primary antibody ezrin was diluted to 1:1500 and added to the slides to be incubated overnight at 4°C. The slides were then washed three times for 5 minutes in TBST before visualization with the "DAKO LSAB2 System, Peroxidase" DAKO A/S, No K0675). Control slides were incubated with secondary antibody only. After washing three times for 5 minutes each in TBST, the slides were mounted and analyzed under microscope by authors blindly.

### Statistical analysis

All data are presented as percentage of patients or mean with standard deviation. Numerical data were compared by independent two-sample t tests. Nominal data were compared by Pearson chi-square test, Fisher exact test or multiple forward stepwise logistic regression test when appropriate. Survival was calculated and plots constructed according to the Kaplan-Meier method. Furthermore, the log-rank test was performed for a statistical univariate analysis of prognostic variables. All statistical analyses were performed using the SPSS computer software package (Version 10.0, Chicago, IL, USA). A value of P ≤ 0.05 was considered significant.

## Results

### Clinicopathological features of 104 HBV HCC patients after hepatectomy regarding ezrin staining status

Table 1 summarized the clinicopathological features of 104 HBV HCC patients, including 86 men and 18 women with a median age of 53.4 years. All the 104 patients were positive for hepatitis B surface antigen. Among them, 67 out of 104 (64.4%) patients had underlying cirrhosis and all the 67 patients had Child A grading. 64 out of 104 patients had high grade HCC (grade III and IV) (61.5%), 66 capsular invasion (63.5%), 25 vascular invasion (24.0%), 27 satellite lesions (26.0%), and 68 recurrence after hepatectomy (65.3%). Regarding staining status of ezrin, the HBV-HCC patients with positive ezrin immunoreactivity had smaller tumor size, higher frequency of cirrhosis, tumor de-differentiation, satellite lesions, and vascular invasion. Multivariate forward logistic regression analysis showed that small tumor size, cirrhotic liver, poor differentiation, and vascular invasion are the four independent factors associated with HBV HCC patients with positive ezrin immunoreactivity (Table 2 and 3).

**Table 1:** Clinicopathological features of 104 patients with hepatitis-B HCC undergoing hepatectomy

Demographic data	
Male: Female	86:18
Age (years) (median; range)	53.4; 23–79
AFP (ng/ml) (median; range)	143; 3–10001
HBsAg positive, (%)	104 (100)
Tumor size (cm) (median; range)	5.0; 1.5–20.5
Liver cirrhosis (+) (%)	67/104 (64.4%)

Child grading	

A	67
B	0

**Extent of hepatic resection**	

Extended right lobectomy	10 (9.6%)
Right lobectomy	17 (16.3%)
Extended left lobectomy	5 (4.8%)
Left lobectomy	13 (12.5%)
Partial hepatectomy	59 (56.7%)

**Pathological features**	

Tumor differentiation (I, II, III, and IV)	3,37,54,10
Capsular invasion	66/104 (63.5%)
Vascular invasion	25/104 (24.0%)
Satellite lesions	27/104 (26.0%)
Recurrence	68/104 (65.4%)

HBsAg: hepatitis B surface antigen

**Table 2:** Clinicopathological features between Ezrin (+) and Ezrin (-) hepatitis-B HCC patients

	E (+) (n = 27) (%)	E (-) (n = 77) (%)	P
Age (years)	51.1 ± 13.3	52.6 ± 13.4	0.631
Gender (M:F)	24:3	62:15	0.323
Tumor size (cm)	5.0 ± 4.3	7.0 ± 4.7	0.049
AFP(ng/ml)	1288.1 ± 2898.0	2215.3 ± 3701.7	0.201
Cirrhosis (+)	23 (85.2)	44 (58.7)	0.013
Grading (Edmonson and Stainer)			0.044
Low-grade (I+ II)	6 (22.2)	34 (44.2)	
High-grade (III+ IV)	21 (77.8)	43 (55.8)	
Capsule formation (+)	16 (58.9)	50 (64.9)	0.642
Vascular invasion (+)	12 (44.4)	13 (16.9)	0.004
Satellite lesions (+)	11 (40.7)	16 (21.3)	0.050
Recurrence (+)	17 (63.0)	51 (66.2)	0.759

M: male; F: female; AFP: α-fetoprotein

**Table 3:** Multiple forward stepwise logistic regression analysis of clinicopathological features in 104 surgical resected hepatitis-B HCC cases (Ezrin (+) and Ezrin (-) group)

	Odds ratio	95% CI for Odds Ratio: Lower-Upper	P
Size<3 cm/>3 cm	3.174	1.083–9.301	0.035
Cirrhosis/Non-cirrhosis	4.464	1.269–15.625	0.020
Edmonson-Steiner			0.027
Gr III+IV/Gr I+II	3.759	1.161–12.195	
Vascular invasion (+)/(-)	4.149	1.314–12.987	0.015
Satellite lesions (+)/(-)			NS

### Positive fluorescent and western blotting of ezrin-vsvg expressed in Hep 3B cells

**Figure 1:**
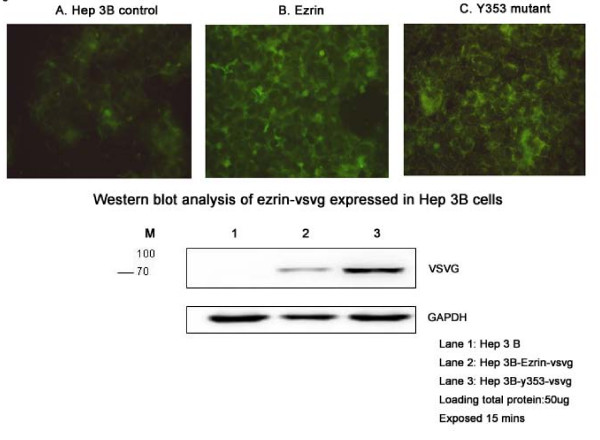
**Upper row: Positive fluorescent staining of VSVG in the wild type (wt) ezrin and Y-353 Hep 3B cells, indicating successful transfection of ezrin into Hep 3B cells**. Lower row: Western blotting of VSVG in the wt-ezrin and Y-353 Hep 3B cells, meaning successful functional expression of ezrin construct in the transfected Hep 3B cells.

Figure 1 upper row illustrated positive fluorescent staining of VSVG in the wt-ezrin and Y-353 Hep 3B cells, indicating successful transfection of ezrin into Hep 3B cells. Figure 1 lower row depicted western blotting of VSVG expression in the wt-ezrin and Y353-ezrin Hep 3B cells, meaning successful functional expression of ezrin construct in the transfected Hep 3B cells.

### Ezrin Is Important for the Matrigel Invasion of Hep 3B Cell Lines

**Figure 2:**
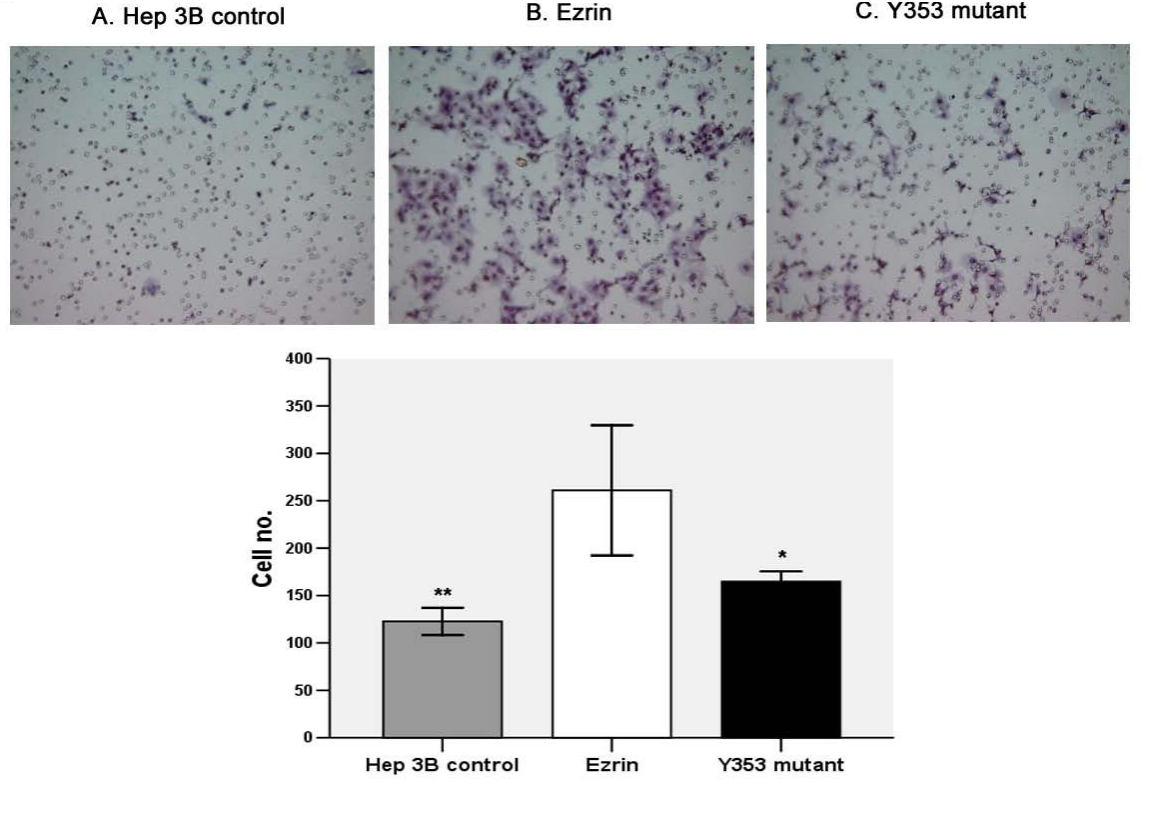
**Wt-ezrin Hep 3B cells had significant higher invasiveness than Hep 3B cells**. However, inhibition of ezrin function using Y353-ezrin mutatnt significantly reduces invasion Hep 3B cells.

We studied the possibility of ezrin being involved in the regulation of the invasive properties of Hep3B. Figure 2 illustrates that ezrin transfected Hep 3B cells have significant higher invasiveness than Hep 3B cells. However, inhibition of ezrin function as shown in theY353-ezrin clones has significantly reduced the invasion of Hep 3B.

### Determination of AFP and ALB concentration in the extracellular medium

**Figure 3:**
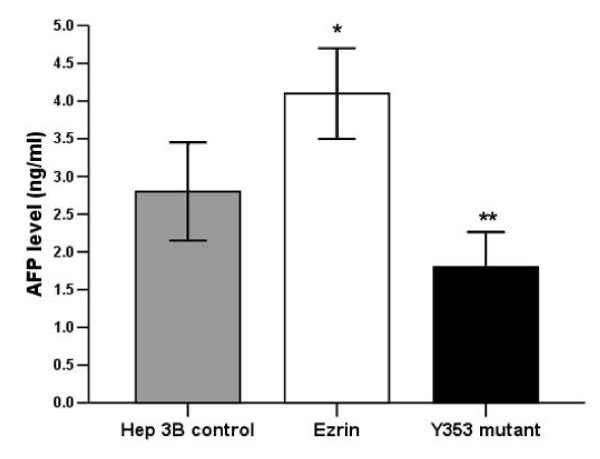
**Wt-ezrin Hep 3B cell secreted significant higher AFP level when compared with Hep 3B cell, indicating ezrin is associated with de-differentiation of Hep 3B cells**. But Y-353 Hep 3B cell secreted significant lower AFP level when compared with wt-ezrin Hep 3B and Hep 3B cells.

AFP and ALB in the extracellular medium is produced and released into extracellular fluids by cells. The appearance of AFP and ALB in the medium further confirms tumor cell de-differentiation and differentiation, respectively. In our experiments, wt-ezrin Hep 3B cell secreted significant higher AFP level when compared with Hep 3B cell, indicating ezrin is associated with de-differentiation of Hep 3B cells. But Y353-ezrin clones secreted significant lower AFP level when compared with wt-ezrin clones and the control Hep 3B cells (Figure 3). Albumin level is too low to be detected.

### Immunohistochemistry

**Figure 4:**
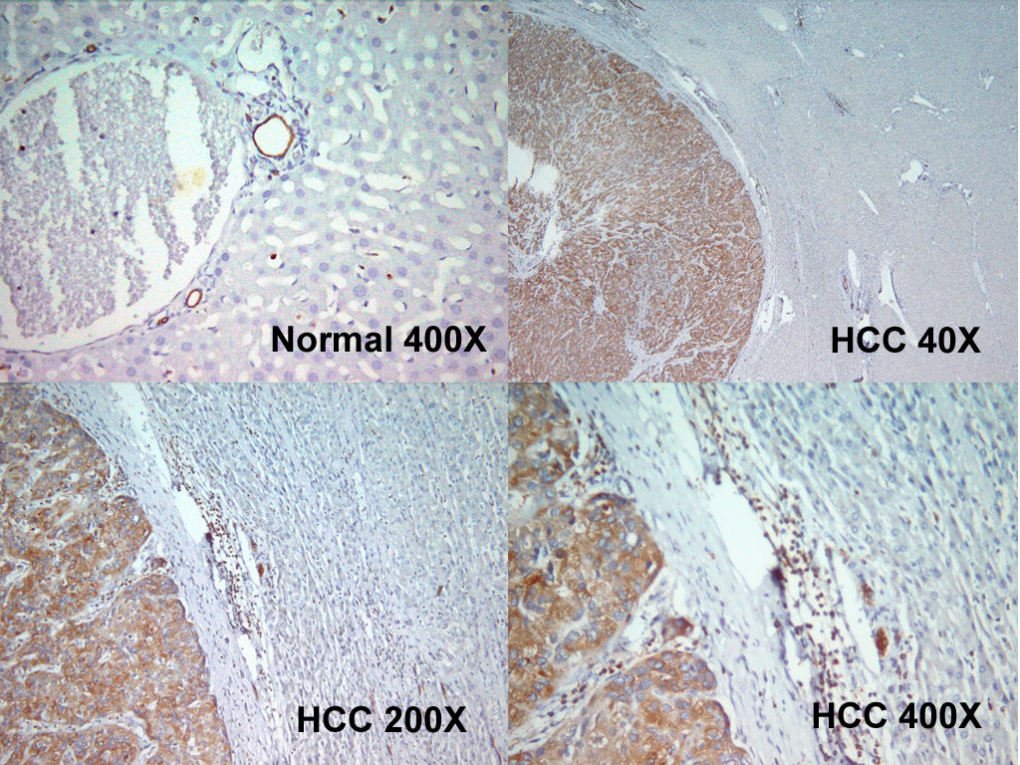
**(A). Ezrin is not stained in the normal hepatocyte but positive in the lymphocyte and cholagiocyte (cholangiocyte and lymphocyte can be used as internal positive control)**. (B, C, D). Positive ezrin immunoreactivity was observed in the membrane and/or cytoplasm of the tumor cells from low power to high power field (B, × 40; C ×200; D ×400).

Figure 4A illustrated that ezrin is not stained in the normal hepatocyte but positive in the lymphocyte and cholangiocyte (cholangiocyte and lymphocyte can be used as internal positive control). Among the 104 HBV HCC patients undergoing hepatectomy, 27 (29.6%) had variable positive ezrin immunoreactivity in the cytoplasm of the tumor cell (Figure 4B, C, and 4D).

### Survival analysis of HBV-HCC patients undergoing hepatectomy in terms of ezrin staining status

All of the 104 HBV-HCC patients undergoing hepatectomy were closely followed at regular intervals until death or until the time of this writing. The median follow-up period of the 104 HBV-HCC patients undergoing hepatectomy is 36.4 months (range: 1.05–134.0 months). Similar disease-free survival (DFS) and overall survival (OS) rate were observed between the ezrin positive and ezrin negative HBV-HCC patients after hepatectomy. The 1-, 3-, and 5-year DFS rates between ezrin positive and ezrin negative HBV-HCC patients after hepatectomy are similar. Furthermore, the 1-, 3-, and 5-year OS rate between the ezrin positive and ezrin negative HBV-HCC patients after hepatectomy are also similar.

## Discussion and Conclusion

Ezrin is a key signaling molecule that regulates cell survival, adhesion, migration, and invasion, which plays a role of linker protein with capacity to bind membrane proteins and the actin cytoskeleton, such interaction provides an intracellular scaffold for the formation of specialized membrane domains that facilitate signal transduction through a number of growth factor receptor and adhesion molecules [21]. The main function of ezrin was first known to interact with p85, the regulatory subunit of PI3-kinase (PI3K), which was involved in determining survival of epithelial cells by activating the PI3K/Akt pathway [13]. Subsequently, the regulation of adhesion, migration and invasion were observed [21] and proved important to tumor development and progression [21]. Although, over-expression of ezrin has been detected in several human epithelial tumors, including brain hemangioblastoma [16], uterine endometrioid adenocarcinoma [17], osteosarcoma [18], and uveal malignant melanoma [19], this is the first study to investigate the role of ezrin on HBV-HCC from cell level to patients with HBV-HCC undergoing hepatectomy.

HBV-HCC patients with positive ezrin immunoreactivity had smaller tumor size, higher frequency of cirrhosis, tumor dedifferentiation, satellite lesions, and vascular invasion. Multivariate forward logistic regression analysis showed that small tumor size, cirrhotic liver, poor differentiation, and vascular invasion are the four independent factors associated with HBV HCC patients with positive ezrin immunoreactivity.

Regarding differentiation, HBV-HCC patients with positive ezrin immunoreactivity had more poor differentiation. In vitro study, we demonstrated increased activity of ezrin resulted in de-differentiation of Hep3B cell as shown increased secretion of AFP and absence of albumin secretion. Recently, the activated PI3K-Akt-mTOR pathway has recently emerged as a novel contributor to HCC development [22]. Furthermore, PI3K-Akt-mTOR pathway is associated with ezrin. Sustained activation of mTOR impaired the hepatocytic differentiation capability of these cells as shown by impaired formation of bile canaliculi, absence of polarity, and reduced secretion of A1-antitrypsin. Decrease in the activity of mTOR observed on hepatocytic differentiation of the HepaRG cell (a cell line established from the nontumoral region of a resected HCV-associated HCC). Increased Akt-mTOR activity contributed to dedifferentiation of HepaRG cells [23]. Similar to HepaRG cells, increased activity of ezrin may increase the activity of PI3K-Akt-mTOR pathway resulting in de-differentiation of Hep3B cell. Therefore, the role of ezrin in HCC differentiation could probably mediate through the pathway of PI3K-Akt-mTOR.

Regarding invasion, increased activity of ezrin contributed to increased invasiveness of Hep3B cell as shown increased invasion of matrigel invasion assay.

On the other hand, decreased activity of ezrin by transfection of dominant-negative mutant (Y353) dramatically decreased the invasion ability of HCC cell line, because Y353 ezrin change the antiapoptotic signal of ezrin to apoptosis [13]. In experimental invasion assay, demonstrating that ezrin over-expression is sufficient to confer metastatic capability. HBV-HCC patients with positive ezrin immunoreactivity had more vascular invasion but no higher rate of recurrence. This contradiction could be explained by HBV-HCC patients with positive ezrin immunoreactivity having smaller tumor size.

Regarding survival, ezrin expression was not an independent prognostic factor for HBV-HCC patients after hepatectomy with long-term disease-free survival and overall survival. Small tumor size, cirrhotic liver, poor differentiation, and vascular invasion are the four independent factors associated with HBV HCC patients with positive ezrin immunoreactivity.

Our previous study has demonstrated that overall survival and disease-free survival for small sized HCC is better than large HCC [24]. Patients with long-term disease-free survival after hepatectomy usually had lower rate of underlying liver cirrhosis. The effect of underlying cirrhosis in the non-tumorous liver on risk of recurrence after HCC resection is, however, controversial, although cirrhosis was reported to be a significant risk factor for recurrence in the remaining liver, owing to a predisposition to multicentric hepatocarcinogenesis [25]. In addition, when the patients with low-grade HCC after hepatectomy was related with the long-term disease-free survival, however, the prognostic significance of histological grading of HCC on risk of recurrence is debatable [26]. Vascular invasion is the most consistently reported risk factor for recurrence after HCC resection [27]. Intrahepatic portal vein involvement is widely accepted as the mechanism for intrahepatic recurrence for HCC. Although our study shows that expression of ezrin is related to tumor differentiation status and vascular invasion ability, its expression has no significant impact on survival for our patient group. In addition, the expression of ezrin has an inversely relationship with the tumor size. This seems to be a conflicting outcome of our analysis, however, as we were looking at a group of patient who received surgical treatment, the result can not truly reflect the impact of this marker on HCC patients. In general, one would expect that, patient with smaller tumor size would have a better outcome if completely resection of the tumor can be made. Taken together, our result of ezrin expression status has no impact on survival for HBV-HCC patients who had undergone hepatectomy has to be carefully interpreted. Therefore, it would be of importance to study the expression of ezrin including other hepatitis related HCC patients in order to establish its role as an independent prognostic marker for HCC.

In conclusion, over-expression of ezrin in HBV-HCC patients was independently associated with small tumor size, cirrhotic liver, poor differentiation, and vascular invasion. Ezrin over-expression contributes to de-differentiation and invasion of HBV-HCC cell.

## Competing interests

The authors declare that they have no competing interests.

## Authors' contributions

**CNY **designed the experiment and drafted the manuscript. **STP **drafted and revised the manuscript and contributed equally to the paper as the first author CNY. **TWC **carried out the transfection, western blotting, and immuohistochemical staining. **RCW **examined the immunostaing of the slide and carried out the scoring of the immuostaing results.**WHW **contributed to the revision and wording of the manuscript. **MFC **assisted and supervised the design of the experiment and draft of the manuscript. All authors read and approved the final version of the manuscript.

## Pre-publication history

The pre-publication history for this paper can be accessed here:

http://www.biomedcentral.com/1471-2407/9/233/prepub
